# PD-1/PD-L pathway inhibits *M*.*tb*-specific CD4^+^ T-cell functions and phagocytosis of macrophages in active tuberculosis

**DOI:** 10.1038/srep38362

**Published:** 2016-12-07

**Authors:** Lei Shen, Yan Gao, Yuanyuan Liu, Bingyan Zhang, Qianqian Liu, Jing Wu, Lin Fan, Qinfang Ou, Wenhong Zhang, Lingyun Shao

**Affiliations:** 1 Department of Thoracic Intensive Care Unit, Shanghai Pulmonary Hospital, Tongji University, Shanghai, 200433, China; 2 Department of Infectious Diseases, Huashan Hospital, Fudan University, Shanghai, 200040, China; 3 Clinic and Research Center of Tuberculosis, Shanghai Pulmonary Hospital, Tongji University, Shanghai, 200433, China; 4 Department of Pulmonary Diseases, Wuxi Infectious Diseases Hospital, Wuxi, 214005, China; 5 Key Laboratory of Medical Molecular Virology, Ministry of Education and Health, Shanghai Medical College, and Institutes of Biomedical Science, Fudan University, Shanghai, 200032, China

## Abstract

The role of the PD-1/PD-L pathway in a murine model of tuberculosis remains controversial regarding viral infections and clinical tuberculosis. We conducted a case-control study to investigate the modulating role and mechanism of the PD-1/PD-L pathway in patients with active tuberculosis. Fifty-nine participants, including 43 active tuberculosis (ATB) patients and 16 healthy controls (HC), were enrolled. Cell surface staining and flow cytometry were used to detect the expressions of PD-1 and its ligands on T cells and monocytes. Intracellular cytokine staining was used to determine the PPD-specific IFN-γ-secreting T-cell proportion. CD4^+^ T-cell proliferation and macrophage functions were investigated in the presence or absence of PD-1/PD-L pathway blockade. Proportions of both PD-1^+^CD4^+^ and PD-L1^+^CD4^+^ T cells in ATB patients were more significantly increased than in the HC group (*P* = 0.0112 and *P* = 0.0141, respectively). The expressions of PD-1, PD-L1, and PD-L2 on CD14^+^ monocytes in ATB patients were much higher than those in the HC group (*P* = 0.0016, *P* = 0.0001, and *P* = 0.0088, respectively). Blockade of PD-1 could significantly enhance CD4^+^ T-cell proliferation (*P* = 0.0433). Phagocytosis and intracellular killing activity of macrophages increased significantly with PD-1/PD-L pathway blockade. In conclusion, the PD-1/PD-L pathway inhibits not only *M*.*tb*-specific CD4^+^ T-cell-mediated immunity but also innate immunity.

*Mycobacterium tuberculosis* (*M*.*tb*), as an intracellular pathogen, causes tuberculosis infection, which is a major global health issue. Approximately 9.6 million new cases occurred in 2014, and 1.5 million people have died from TB^1^. Approximately 5–10% of infected people develop TB during their lifetime when the host immune response fails to maintain *M*.*tb* in a latent form and the risk is increased by a compromised immune system^2^.

The immune system protects the host by combating *M*.*tb*, but it also has to regulate this response to curtail tissue damage. Regulatory T cells (Tregs) and the PD-1/PD-L pathway are both critical for terminating the immune responses^3^,^4^,^5^. Other co-inhibitory molecules, such as cytotoxic T lymphocyte antigen-4 (CTLA-4)^6^, T cell immuoglobulin and mucin domain-3 (Tim-3)^7^ have also been reported to influence the delicate balance between protective immunity and tolerance.

The PD-1/PD-L pathway includes PD-1 (also known as CD279) and its two ligands: PD-L1 (also known as CD274 and B7-H1) and PD-L2 (also known as CD273 and B7-DC), which are type I transmembrane proteins belonging to the CD28 superfamily. PD-1 was first discovered in 1992; its gene is upregulated in the programmed death of T-lymphocyte hybridoma^8^. PD-1 and its role in T-cell exhaustion were initially determined in microarray studies that screened the gene expression difference in murine CD8^+^ T cells between acute and chronic lymphocytic choriomeningitis virus (LCMV) infection^9^. In human virus infection, such as human immunodeficiency virus (HIV)^10^ and hepatitis C virus (HCV)^11^, the PD-1 pathway mediates T-cell exhaustion, which can be restored by blocking the PD-1 pathway. Overexpression of PD-1 and its ligands on T cells, NK cells, and macrophages of patients with tuberculosis as well as its inhibitory role in innate and adaptive immunity have been reported^12^,^13^. However, the exact mechanism of inhibiting the T-cell response and macrophage function against *M*.*tb* has yet to be elucidated.

Thus, we conducted a case-control study using an *in vitro* system to investigate the modulating role and mechanism of the PD-1/PD-L pathway in active tuberculosis.

## Results

### Clinical characteristics of enrolled participants

The diagnosis of active tuberculosis (ATB) was established based on clinical symptoms, radiological data, and identification of acid-fast bacilli in sputum or pleural effusion, or was clinically confirmed by anti-TB therapy. The 59 enrolled participants were divided into two groups: the ATB group (*n* = 43), which included patients with confirmed active pulmonary tuberculosis (*n* = 19) and tuberculosis pleurisy (*n* = 24), and the healthy control (HC) group (*n* = 16).

In the ATB group, patients with confirmed pulmonary tuberculosis (*n* = 19) were all culture-positive for *M*.*tb* in sputum or bronchoalveolar lavage fluid (BALF). Patients with tuberculosis pleurisy (*n* = 24) were culture-positive for *M*.*tb* in the pleural fluid (*n* = 3) and in the sputum (*n* = 6), and were clinically confirmed by anti-TB therapy (*n* = 16). The average age of the ATB group was 40.9 years; 33 out of 43 participants were male (76.7%). Twenty-six (60.5%) of 43 participants received TB treatment. The treatment duration at enrollment was no more than 2 wk. Three out of 43 (7%) patients had a history of exposure to active tuberculosis. In the HC group, the average age was 32.4 years; 9 out of 16 participants were male (56.3%). Five (31.3%) had a history of exposure to ATB. The characteristics of the three groups were described in Table 1. All enrolled participants were HIV-negative, had not been diagnosed with cancer, diabetes, autoimmune diseases or other chronic infections (i.e., chronic HBV/HCV infection), and had not received immune modulator treatments.

The patients were examined after they provided informed consent. Peripheral blood samples were collected in heparinized tubes and pleural effusion samples were obtained by thoracentesis.

### Increased expressions of PD-1 and its ligands on CD4^+^ T cells and CD14^+^ monocytes, but not on CD8^+^ T cells, among patients with active tuberculosis

**Figure 1:**
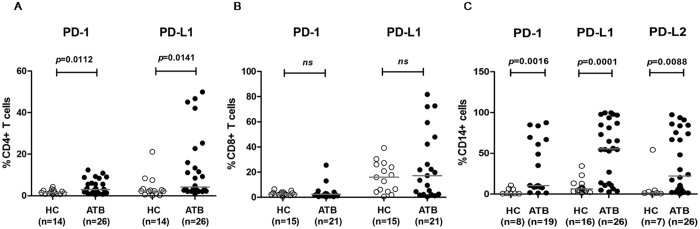
Expressions of PD-1 and its ligands on CD4^+^ and CD8^+^ T cells and CD14^+^ monocytes. (**A**) Expressions of PD-1 and PD-L1 on CD4^+^ T cells in the ATB group were increased significantly compared to the HC group (*P* = 0.0112 and *P* = 0.0141, respectively). (**B**) Expressions of PD-1 and PD-L1 on CD8^+^ T cells in the ATB group were similar with the HC group. (**C**) Expressions of PD-1, PD-L1, and PD-L2 on CD14^+^ monocytes in the ATB group were all increased remarkably compared to that in the HC group (*P* = 0.0016, *P* = 0.0001, and *P* = 0.0088, respectively).

We first detected the expressions of PD-1/PD-L on T-cell subsets. As expected, the frequency of PD-1-expressing CD4^+^ T cells in ATB patients was significantly higher than in healthy controls (*P* = 0.0112). The expression of PD-L1 on CD4^+^ T cells in ATB patients was also higher than that in healthy controls (*P* = 0.0141) (Fig. 1A). However, upregulations of PD-1 and PD-L1 in ATB patients were not observed on CD8^+^ T cells (Fig. 1B). Because PD-1-mediated inhibition of effector T-cell functions required engagement with the expression of its ligands on APCs, we further examined the expressions of PD-1 and its ligands on CD14^+^ monocytes. Significantly greater increases of PD-1, PD-L1, and PD-L2 on CD14^+^ monocytes were observed in ATB patients compared to those in HC (*P* = 0.0016, *P* = 0.0001, and *P* = 0.0088, respectively) (Fig. 1C). The representative flow cytometry analysis was shown in Supplementary Figure S1.

### Frequency of *M.tb*-specific IFN-γ-secreting CD4^+^ T cells

**Figure 2:**
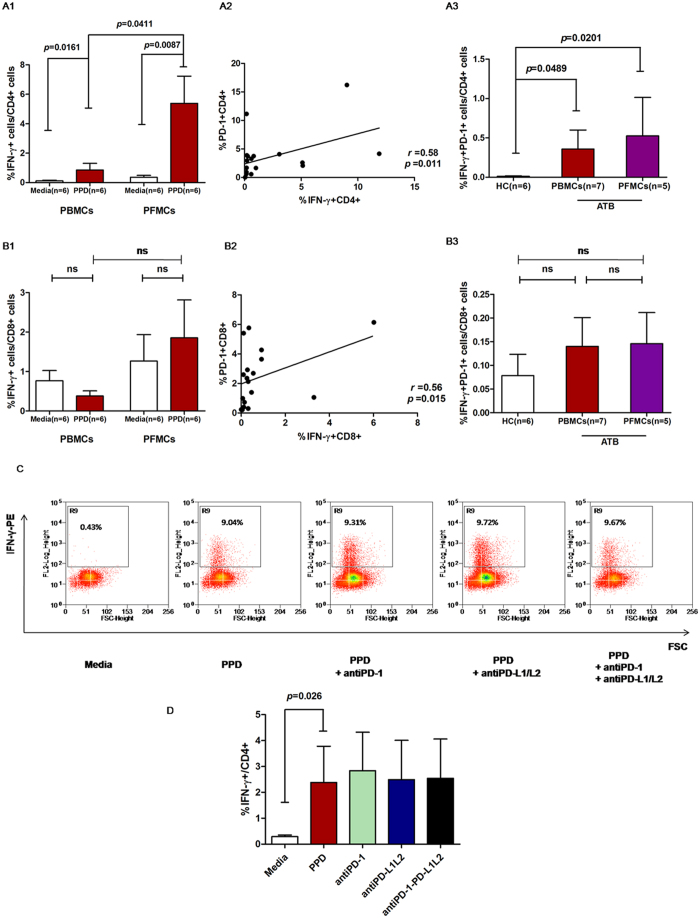
The influence of the PD-1/PD-L pathway on *M*.*tb*-specific IFN-γ secretion. (**A1**) IFN-γ-secreting CD4^+^ T cells increased in both PBMCs and PFMCs of ATB patients stimulated with PPD (*P* = 0.0161 and *P* = 0.0087). Those in PFMCs were higher than those in PBMCs (*P* = 0.0411). (**A2**) The correlation between PD-1 expression and IFN-γ secretion of CD4^+^ T cells when stimulated with PPD was significant (*r* = 0.58; *P* = 0.011). (**A3**) Expression of PD-1 in IFN-γ-secreting CD4^+^ T cells stimulated with PPD in PBMCs and PFMCs of ATB group was both higher than that in PBMCs of the HC group (*P* = 0.0489 and *P* = 0.0201, respectively). (**B1**) The frequency of IFN-γ-secreting CD8^+^ T cells in PBMCs and PFMCs of ATB patients stimulated with PPD was similar. (**B2**) The correlation between PD-1 expression and IFN-γ secretion of CD8^+^ T cells when stimulated with PPD was significant (*r* = 0.56, *P* = 0.015). (**B3**) The expression of PD-1 in IFN-γ-secreting CD8^+^ T cells stimulated with PPD in PBMCs and PFMCs of the ATB group was similar to that in PBMCs of the HC group. (**C**) Representative CD4-gated histograms of PPD-specific IFN-γ secretion blocked with PD-1/PD-L pathway Abs. (**D**) After blocking of the PD-1/PD-L pathway with anti-PD-1 and/or anti-PD-L1/PD-L2 Abs, the IFN-γ secretion of CD4^+^ T cells was not significantly different from each other.

We further stimulated the peripheral blood mononuclear cells (PBMCs) and pleural fluid mononuclear cells (PFMCs) with purified protein derivative (PPD) and examined IFN-γ secretion to investigate the frequency of *M*.*tb*-specific IFN-γ-secreting T cells. As expected, the frequency of *M*.*tb-*specific IFN-γ-secreting CD4^+^ T cells stimulated with PPD in the peripheral blood samples of ATB patients was significantly increased compared with media only (*P* = 0.0161; Fig. 2A1). We also checked the frequency of PPD-specific IFN-γ-secreting CD4^+^ T cells in PFMCs from tuberculosis pleurisy patients and found that the IFN-γ-secreting CD4^+^ T cells frequency in PFMCs from tuberculosis pleurisy patients increased significantly when stimulated with PPD (*P* = 0.0087; Fig. 2A1). And it also increased significantly when compared with stimulated PBMCs from ATB patients (*P* = 0.0411; Fig. 2A1). However, these changes were not observed on CD8^+^ T cells in PBMCs and PFMCs (Fig. 2B1).

To understand the correlation between the change in IFN-γ secretion and the PD-1 expression, we simultaneously analyzed IFN-γ secretion and PD-1 expression both on CD4^+^ and CD8^+^ T cells. We found that there was a significant positive correlation between PD-1 expression and IFN-γ secretion on CD4^+^ and CD8^+^ T cells when stimulated with PPD (for CD4^+^ T cells, *r* = 0.58, *P* = 0.011, Fig. 2A2 and for CD8^+^ T cells, *r* = 0.56, *P* = 0.015, Fig. 2B2). We further found that the expression of PD-1 on IFN-γ-secreting CD4^+^ T cells in the peripheral blood samples of ATB patients and in the pleural fluid of tuberculosis pleurisy patients was both higher than that in HC group (*P* = 0.0489 and *P* = 0.0201; Fig. 2A3). However, PD-1 expression on IFN-γ-producing CD8^+^ T cells in the peripheral blood samples of ATB patients and in the pleural fluid of tuberculosis pleurisy patients was similar with HC group (Fig. 2B3). These data suggested that PD-1, as an inhibitory molecule, mainly modulated CD4^+^ T cells rather than CD8^+^ T cells in tuberculosis.

Next, we used specific PD-1/PD-L pathway blockade antibodies (Abs) to investigate the role of PD-1/PD-L pathway in IFN-γ secretion of CD4^+^ T cells. Disappointingly, as shown in Fig. 2C and D, the IFN-γ secretion of CD4^+^ T cells increased remarkably after PPD stimulation, but did not change significantly after PD-1/PD-L were blocked alone or combined. These results indicate that besides influencing the IFN-γ secreting, there might be other mechanisms responsible for PD-1/PD-L pathway in the pathogenesis of tuberculosis.

### Blockade of the PD-1/PD-L pathway enhanced the proliferation of *M.tb*-specific CD4^+^ T cells *in vitro*

**Figure 3:**
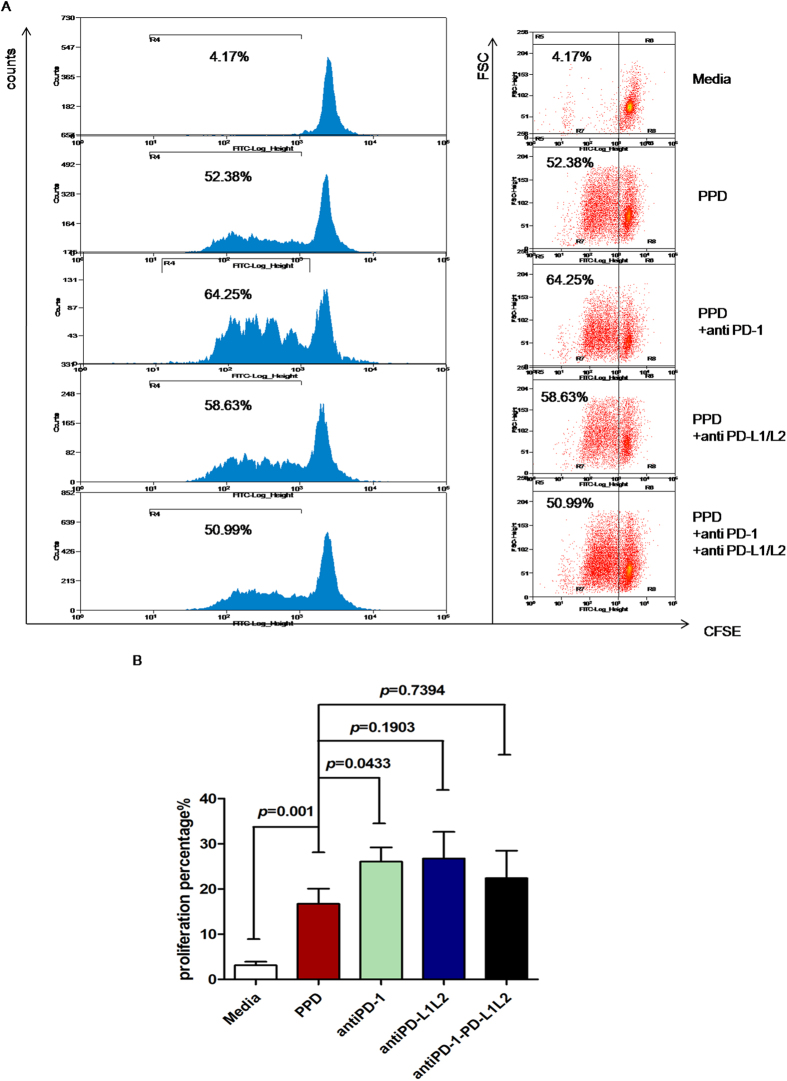
Inhibition of PD-1/PD-L pathway enhanced *M*.*tb*-specific CD4^+^ T-cell proliferation *in vitro*. (**A**) Representative CD4-gated histograms (left) and dot plots (right) of CFSE proliferation assay *in vitro*. The percentage of proliferative CD4^+^ T cells cultured with media was only 4.17%. When stimulated with PPD/CD3/CD28, the proliferative percentage increased as high as 52.38%. The percentage increased to 64.25% when blocked with anti-PD-1 Ab. The percentage was 58.63% when blocked with both anti-PD-L1 and anti-PD-L2, and 50.99% when blocked simultaneously with anti-PD-1, anti-PD-L1 and anti-PD-L2. (**B**) CD4^+^ T-cell proliferation stimulated with PPD was higher than stimulated with media (*P* = 0.001), and proliferation in the anti-PD-1 group was higher than in the PPD group (*P* = 0.0433). There were no remarkable differences in the anti-PDL1/L2 group and combined blockade group compared to the PPD group.

Because *M*.*tb*-specific IFN-γ secretion did not change significantly by blocking the PD-1/PD-L pathway, we further tested the effect of blockade of the PD-1/PD-L pathway on *M*.*tb* antigen-induced CD4^+^ T-cell proliferation using a flow cytometry based CFSE diluted assay. The representative histograms (left) and dot plots (right) flow analysis were shown in Fig. 3A. Interestingly, we observed a significant increase in CD4^+^ T-cell proliferation in the presence of PPD (*P* = 0.001) and PPD with PD-1 blockade (*P* = 0.0433). The increasing tendency in the presence of PD-L blockade alone or together with PD-1 blocking Ab was shown, but the difference was not significant (*P* = 0.1903 and *P* = 0.7394, respectively) (Fig. 3B).

### Inhibition of the PD-1/PD-L pathway helped to control Mycobacterium bovis bacillus Calmette-Guerin (BCG) replication in macrophages co-cultured with CD4^+^ T cells *in vitro*

**Figure 4:**
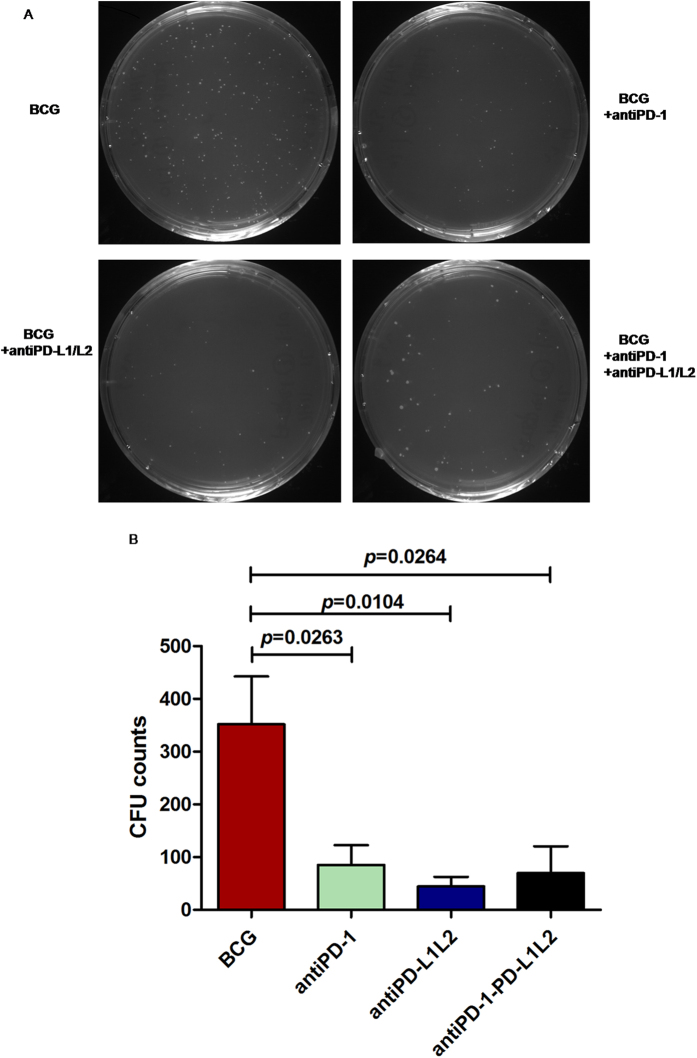
Inhibition of PD-1/PD-L pathway helped to control BCG replications when MDMs co-cultured with CD4^+^ T cells *in vitro*. (**A**) Representative BCG CFU counts with different treatments. MDMs were infected with BCG (MOI = 10) for 4 h and then co-cultured with CD4^+^ T cells (MDMs/CD4 = 1:10) for 7 d. Whole-cell lysate was diluted and aliquots of each dilution of lysate were plated onto 7H11 agar and incubated for 3 wk at 37 °C until colonies were large enough to be counted. MDMs were prepared with the presence or absence of anti-PD-L1 and anti-PD-L2 Abs for 1 h before infection and CD4^+^ T cells were prepared with the presence or absence of anti-PD-1 Ab for 1 h before co-culture. (**B**) CD4^+^ T cells blocked with PD-1 Ab significantly inhibited the intracellular BCG replication in MDMs (*P* = 0.0263). Additionally, MDMs blocked with PD-L1/L2 Abs significantly inhibited the intracellular BCG replications (*P* = 0.0104). Blocking CD4^+^ T cells with PD-1 Ab and MDMs with PD-L1/L2 Abs similarly inhibited BCG replications in MDMs (*P* = 0.0264).

To test the effect of the PD-1/PD-L pathway blockade toward BCG replication in monocyte-derived macrophages (MDMs) controlled by T cells, we infected MDMs with BCG [multiplicity of infection (MOI) = 10] and co-cultured with CD4^+^ T cells (1:10). MDMs and T cells were pretreated for 1 h with or without PD-1 and PD-L1/L2 blocking Abs according to different experiment designs. Interestingly, CD4^+^ T cells blocked with PD-1 antibody significantly inhibited the intracellular BCG replication in MDMs (*P* = 0.0263). Additionally, MDMs blocked with PD-L1/L2 Abs significantly inhibited the intracellular BCG replication (*P* = 0.0104). Furthermore, simultaneously blocking CD4^+^ T cells with PD-1 Ab and MDMs with PD-L1/L2 Abs similarly inhibited BCG replication in MDMs (*P* = 0.0264) (Fig. 4A and B).

## Discussion

Impaired immunity in tuberculosis is associated with impaired macrophage and T-cell activation and reduced production of IFN-γ^14^. The inhibitory role of the PD-1/PD-L pathway has been demonstrated in tumors^15^,^16^ and chronic viral infections, such as LCMV, HIV, HCV, and HBV^17^. Blockade of the PD-1/PD-L pathway can partly or completely restore effector T-cells. However, the role of the PD-1/PD-L pathway in murine TB remains controversial according to data of clinical TB and viral infections. A recent study has shown that the survival of PD-1 KO mice after aerosol infection with *M*.*tb* is severely reduced compared to wild-type mice, and a significantly higher bacterium load was observed in the lung and spleen of PD-1 KO mice^18^. Another study has demonstrated that PD-1-deficient mice have a large number of proliferating CD4^+^ T cells in their lungs, thus promoting tuberculosis rather than controlling it^19^. Why the discordance exists between murine and human models has yet to be elucidated. A study of the involved mechanisms has shown an increased frequency of Tregs and reduced T/B-cell proliferation in PD-1 KO mice, indicating that PD-1 plays a protective role in murine TB^20^. However, clinical studies of overexpression of PD-1 and its ligands in ATB patients as well as its inhibitory role in adaptive and innate immunity have been reported^12^,^13^,^21^. Here, we present more information regarding the inhibitory role of the PD-1/PD-L pathway in tuberculosis.

Several studies have demonstrated that the PD-1/PD-L pathway inhibited proliferation and adhesion of CD4^+^ T cells^22^, and that the PD-1/PD-L1 pathway impaired the Th1 immune response during late-stage infection with BCG^23^. Our study showed that proportions of PD-1^+^CD4^+^ and PD-L1^+^CD4^+^ T cells in ATB patients were increased, whereas the expressions of PD-1 and PD-L1 on CD8^+^ T cells were similar between the ATB and HC groups. Because the expression pattern of PD-L2 was limited (expressed on dendritic cells, monocytes, and some B cells on stimulation)^24^, we could not determine PD-L2 expression on CD4^+^ T and CD8^+^ T cells. Our finding was consistent with the results of a gene and protein study showing that PD-1 and PD-L1 expression on CD8^+^ T cells was similar in TB patients and in household contacts^25^. At the local infection site, the expressions of PD-1 and its ligands in pleural fluid of tuberculosis pleurisy were similar to those in peripheral blood (see Supplementary Figure S2), whereas the IFN-γ-producing CD4^+^ T cells at the local infection site increased significantly compared to that in peripheral blood. These changes were not seen in CD8^+^ T cells. These data were consistent with our previous study in which we found that the role of *M*.*tb*-specific CD4^+^ T cells in ATB was more important than CD8^+^ T cells^26^. These results indicated that the inhibitory role of PD-1 which targeted CD4^+^ T cells rather than CD8^+^ T cells in ATB was different from that in viral infections^9^.

In tuberculosis, CD4^+^ T-cell-mediated Th1 response contributes to the main host immunity against *M*.*tb*. The higher expression of PD-1 on CD4^+^ T cells may correlate with T-cell exhaustion. Our further investigation suggested that blockade of PD-1 could significantly augment CD4^+^ T-cell proliferation, but the frequency of IFN-γ-secreting T cells was similar in the presence or absence of blocking Abs. In addition, cytokine level detection showed that IL-2 increased significantly regardless of blocking PD-1 or PD-L1 and PD-L2, but the concentrations of IFN-γ and other cytokines (IL-4, IL-6, IL-10, and TNF-α) were still similar in the presence or absence of the PD-1 pathway blockade (see Supplementary Figure S3). PD-1 expression was confirmed to be upregulated by *M*.*tb*-induced IFN-γ secretion^12^,^13^, and blocking the PD-1/PD-L interaction could enhance *M*.*tb*-specific IFN-γ production by CD3^+^ T cells^21^. However, our study showed that blockade of the PD-1/PD-L pathway did not contribute to IFN-γ secretion. This controversy may be partly explained by the addition of anti-CD28 stimulation. PD-1 can inhibit T-cell receptor activation signal transduction and cause IL-2 withdrawal, and this inhibition can be overcome by IL-2 and CD28.

We also found that the expressions of PD-1 and its ligands on macrophages were much higher among ATB patients, which was in accordance with the results of another study^21^. We further investigated the role of PD-1 and its ligands on the function of macrophages against *Mycobacterium*. Interestingly, we found that phagocytosis (see Supplementary Figure S4) and intracellular killing activity of macrophages against BCG increased significantly when blocking the PD-1/PD-L pathway. Elizabeth *et al*. also demonstrated that PD-L2 induction in dendritic cells exposed to *Mycobacterium avium* downregulated BCG-specific T-cell response^27^. These data suggested that the PD-1/PD-L pathway can exert an inhibitory role on macrophages function. According to our results, the phagocytosis of MDMs was enhanced only when both PD-1 and PD-L1/L2 were blocked together. The possible reasons for the discordance with the mono-antibody blockade of PD-1 or PD-L1/L2 were not clear. It was partly because that PD-L1 and PD-L2 expressed constitutively on dendritic cells and monocytes and took an important role on the innate immunity, such as phagocytosis of pathogens and presenting specific antigens to the adaptive immunity. Ideally, blockade of PD-L1 and PD-L2 could enhance the phagocytosis of MDMs, whereas in our study the assistance of blockade of PD-1 simultaneously was needed to phagocytize the BCG effectively. Although we did not determine the expression of PD-L1/L2 on dendritic cells, it was likely that blocking PD-L1/L2 on adherent cells would block PD-L1/L2 on myeloid dendritic cells, as well as on MDMs^21^. In addition, our study showed that the decreases of PD-L1 and PD-L2 expressions on macrophages were associated with successful treatment of ATB (see Supplementary Figure S2). Thus, all these results indicate the role and underlying mechanism of the PD-1/PD-L pathway on macrophages in tuberculosis and deserve further investigation.

This study had some limitations. As we know that high levels of costimulation could overcome PD-1 mediated inhibitory function, whereas we employed CD28 or CD3 antibodies as costimulator to investigate the IFN-γ expression and CD4^+^ T-cell proliferation. This may attenuate the results to some extent. To minimize the influence of these cross linking antibodies, we set control group to investigate the stimulatory or inhibitory function of T cells in this study.

In conclusion, the PD-1/PD-L pathway inhibits not only *M*.*tb*-specific CD4^+^ T-cell-mediated immunity but also innate immunity, such as phagocytosis of macrophages in ATB. Monitoring PD-1 expression and manipulating the PD-1/PD-L pathway may help to evaluate and restore host protective responses during BCG vaccination and anti-TB treatment^28^.

## Materials and Methods

### Study population

Fifty-nine individuals were recruited for this study, including active tuberculosis (ATB) patients (*n* = 43) and healthy controls (HC) (*n* = 16). The ATB patients were recruited from Wu Xi No. 5 People’s Hospital and Shanghai Pulmonary Hospital from January 1, 2014 to December 31, 2014. Healthy controls were recruited from volunteers at Fudan University during the same period.

This study was approved with written consent by the Ethics Committee of Huashan Hospital, Fudan University with the approval number of 2011-247, and written informed consent was obtained from all the participants. All the methods were carried out in accordance with the approved guidelines and all the experimental protocols were approved by the Human Research Ethics Committee Huashan Hospital, Fudan University.

### Expressions of PD-1, PD-L1, and PD-L2 on T Cells and Monocytes

PBMCs and PFMCs were isolated from heparinized venous blood and pleural fluid by Ficoll density-gradient centrifugation and cultivated in 96-well plates (10^6^ cells per well) with RPMI-1640 (Invitrogen, Carlsbad, CA, USA) medium and completed with 0.3 mg/mL L-glutamine, 5 mM HEPES buffer, 100 μg/mL gentamycin, and 10% inactivated fetal bovine serum (FBS). To evaluate PD-1, PD-L1, and PD-L2 expressions on T cells and monocytes/macrophages, freshly isolated PBMCs and PFMCs were incubated with the following Abs: APC-labeled anti-CD3, FITC-labeled anti-CD3, PB-labeled anti-CD4, PeCy7-labeled anti-CD8 (all these Abs were purchased from BD Bioscience, San Diego, CA, USA), PercpCy5.5-labeled anti-CD14, APC-labeled anti-PD-1, FITC-labeled anti-PD-1, PeCy7-labeled anti-PD-L1, PE-labeled anti-PD-L1 and PE-labeled anti-PD-L2 (all these Abs were purchased from eBioscience, San Diego, CA, USA). Stained samples were fixed with 2% PFA and acquired on a MoFlo XDP flow cytometer (Beckman Coulter, Brea, CA, USA). Data were analyzed with summit 5.2 (Beckman Coulter).

### Intracellular cytokine staining

In separate experiments, freshly isolated cells were seeded in 96-well round plates and stimulated with PPD (25 μg/mL; Mycos Research LLC, Loveland, CO, USA), anti-CD28 (0.5 μg/mL; BD Bioscience) and anti-CD49d (0.5 μg/mL; BD Bioscience) for 1 h. Then, Golgi plugs (BD Bioscience) were added and samples were incubated for an additional 5 h. Cells were then collected and stained with FITC-labeled anti-CD3, PB-labeled anti-CD4, PeCy7-labeled anti-CD8, and APC-labeled anti-PD-1, followed by fixation and permeabilization for subsequent intracellular staining with PE-labeled anti-IFN-γ. The percentage of IFN-γ-secreting cells was determined by flow cytometry. In another experiment, cells with or without blocking Abs against PD-1 (10 μg/mL, J116; eBioscience) and/or PD-L1 (10 μg/mL, MIH1; eBioscience), PD-L2 (10 μg/mL, MIH18; eBioscience) were incubated for 1 h, followed by the addition of PPD (25 μg/mL; Mycos Research LLC), anti-CD28 (0.5 μg/mL, BD Bioscience), and anti-CD49d (0.5 μg/mL, BD Bioscience) as stimulators for another 1 h. Then, Golgi plugs were added and samples were incubated for an additional 5 h. Cells were then stained with FITC-labeled anti-CD3, PB-labeled anti-CD4, PeCy7-labeled anti-CD8, and APC-labeled anti-PD-1, followed by fixation and permeabilization for subsequent intracellular staining with PE-labeled anti-IFN-γ. The percentage of IFN-γ-secreting cells was determined by flow cytometry.

### Enrichment of CD4^+^ T cells and CD14^+^ MDMs

Enriched CD4^+^ T cells were obtained by negative selection with magnetic beads (Miltenyi Biotec, Bergisch Gladbach, Germany). The purity of the cell fractions was evaluated by flow cytometry (>95% for CD4^+^ T cells). CD14^+^ MDMs were obtained from adherent cells. Briefly, the PBMCs were seeded on a 96-well flat-bottom plate and cultured for 4 d. Then, supernatants were removed and washed once with phosphate-buffered saline (PBS). Adherent cells were considered CD14^+^ MDMs.

### T-cell proliferation

T-cell proliferation assays were based on CFSE dilution assays. Enriched CD4^+^ T cells were prepared as described, labeled with 2.5 μM CFSE (Invitrogen), and adjusted to 1 × 10^6^/mL. The 10^5^ CFSE-labeled T cells per well were then treated with or without blocking Abs: anti-PD-1 (10 μg/mL, J116; eBioscience), anti-PD-L1 (10 μg/mL, MIH1; eBioscience), and anti-PD-L2 (10 μg/mL, MIH18; eBioscience) for 1 h. After that, PPD (25 μg/mL; Mycos Research LLC), anti-CD28 (2.5 μg/mL; BD Bioscience), and anti-CD3 (2.5 μg/mL; BD Bioscience) were added simultaneously and cultured in 5% CO_2_ at 37 °C. On day 4, cells were stained with PB-labeled anti-CD4 and then evaluated via dilution of CFSE on flow cytometry. Percentages of cells with diluted CFSE were determined in gated populations of total CD4^+^ T cells.

### Intracellular Mycobacterium growth assay *in vitro*

CD14^+^ MDMs (adherent cells) were prepared as described. The blocking Abs of PD-1 (10 μg/mL, J116; eBioscience), PD-L1 (10 μg/mL, MIH1; eBioscience), and PD-L2 (10 μg/mL, MIH18; eBioscience) were then added for 1 h of incubation before BCG infection. Then, BCG was added at a MOI of 10 after counting the adherent cells. After 4 h of incubation, supernatants were aspirated and MDMs were co-cultured with autologous CD4^+^ T cells at a proportion of 1:10. CD4^+^ T cells were blocked with or without PD-1 Ab (10 μg/mL, J116; eBioscience) for 1 h before co-culture. After being cultured in 5% CO_2_ at 37 °C for 7 d, lysis buffer (0.025% SDS diluted with PBS) was added to each well. Plates were placed on a vortex to lyse cells adequately for 5 min. Lysates from each well were then diluted with PBS at 10-fold, 100-fold, and 1000-fold. Aliquots of each dilution of lysate were plated onto 7H11 agar and incubated for 3 wk at 37 °C until colonies were large enough to be counted.

### Statistical analysis

Results were graphed and analyzed using Prism 5.0 (GraphPad, San Diego, CA, USA). Comparisons between groups were made using unpaired *t* tests or Mann-Whitney tests (when the variances were significantly different). Significance was determined when *P* < 0.05.

## Additional Information

**How to cite this article**: Shen, L. *et al*. PD-1/PD-L pathway inhibits *M.tb*-specific CD4^+^ T-cell functions and phagocytosis of macrophages in active tuberculosis. *Sci. Rep.* **6**, 38362; doi: 10.1038/srep38362 (2016).

**Publisher's note:** Springer Nature remains neutral with regard to jurisdictional claims in published maps and institutional affiliations.

## Supplementary Material

Supplementary Information

## Figures and Tables

**Table 1:** Characteristics of enrolled participants.

	HC	ATB		
		Pulmonary tuberculosis	Tuberculosis pleurisy	Total
n	16	19	24	43
Gender, M/F	9/7	14/5	19/5	33/10
Average age, years (range)	32.4 (24–62)	43.5 (21–72)	38.9 (18–61)	40.9 (18–72)
BCG vaccination history, n (%)	16 (100)	17 (89.5)	21 (87.5)	38 (88.4)
History of exposure to active TB, n (%)	5 (31.3)	2 (10.5)	1 (4.2)	3 (7.0)
Previous TB treatment, n (%)	NA	13 (68.4)	13 (54.2)	26 (60.5)
Sputum smear or culture positive, n (%)	NA	19 (100)	6 (25)	25 (58.1)
Pleural effusion smear or culture positive, n (%)	NA	NA	3 (12.5)	3 (7.0)
