# Supplementary material for: PD-1/PD-L pathway inhibits M.tb-specific CD4+ T-cell functions and phagocytosis of macrophages in active tuberculosis
Source: Sci Rep. 2016 Dec 7;6:38362. doi: 10.1038/srep38362 (PMC5141449; doi:10.1038/srep38362)
Supplement: Supplementary Information [file srep38362-s1.doc]

**Supplementary information**

**PD-1/PD-L pathway inhibits *M.tb*-specific CD4^+^ T-cell functions and phagocytosis of macrophages in active tuberculosis**

Lei Shen^1,2^, Yan Gao^2^, Yuanyuan Liu^2^, Bingyan Zhang^2^, Qianqian Liu^2^, Jing Wu^2^, Lin Fan^3^, Qinfang Ou^4^, Wenhong Zhang^2,5^, Lingyun Shao^2*^

^1^ Department of Thoracic Intensive Care Unit, Shanghai Pulmonary Hospital, Tongji University, Shanghai, 200433, China.

^2^ Department of Infectious Diseases, Huashan Hospital, Fudan University, Shanghai, 200040, China.

^3^ Clinic and Research Center of Tuberculosis, Shanghai Pulmonary Hospital, Tongji University, Shanghai, 200433, China.

^4^ Department of Pulmonary Diseases, Wuxi Infectious Diseases Hospital, Wuxi, 214005, China.

^5^ Key Laboratory of Medical Molecular Virology, Ministry of Education and Health, Shanghai Medical College, and Institutes of Biomedical Science, Fudan University, Shanghai 200032, China.

**Supplementary Materials and Methods**

**Detection of cytokine secretions**

PBMCs and PFMCs were incubated in 96-well round plates (10^6^ cells per well) in RPMI-1640 (Invitrogen) medium, with or without blocking Abs against PD-1 (10 μg/mL, J116; eBioscience), and/or PD-L1 (10 μg/mL, MIH1; eBioscience) and PD-L2 (10 μg/mL, MIH18; eBioscience). One hour later, PPD (25 μg/mL; Mycos Research LLC), anti-CD28 (0.5 μg/mL; BD Bioscience) and anti-CD49d (0.5 μg/mL; BD Bioscience) were added as stimulators for another 72 h. Cells without blocking Abs and with PPD stimulations were used as controls. Then, supernatants were collected and cytokines, including IL-2, IL-4, IL-6, IL-10, IFN-γ, and TNF-α, were measured by multiplexed bead-based cytokine immunoassays (Millipore cytokine assay kit, Darmstadt, Germany).

**Phagocytosis of *Mycobacterium* by MDMs *in vitro***

CD14^+^ MDMs (adherent cells) were prepared as described. The blocking Abs of PD-1 (10 μg/mL, J116; eBioscience), PD-L1 (10 μg/mL, MIH1; eBioscience), and PD-L2 (10 μg/mL, MIH18; eBioscience) were then added for 1 h of incubation before BCG infection according to different designs in separate experiments. Then, BCG or GFP-labeled BCG was added at a MOI of 10 after counting the adherent cells. After 4 h of incubation, supernatants were aspirated and cells were washed three times to remove noningested BCG. Cells were stained with DAPI (100 ng/mL) and fixed with 4% PFA. Then, they were scanned by Cellomics ArrayScan VTI HCS Reader (Thermo Scientific, Waltham, MA, USA). The percentage of MDMs that BCG was ingested was calculated to determine the phagocytosis function of MDMs.

**Supplementary Figures**

**
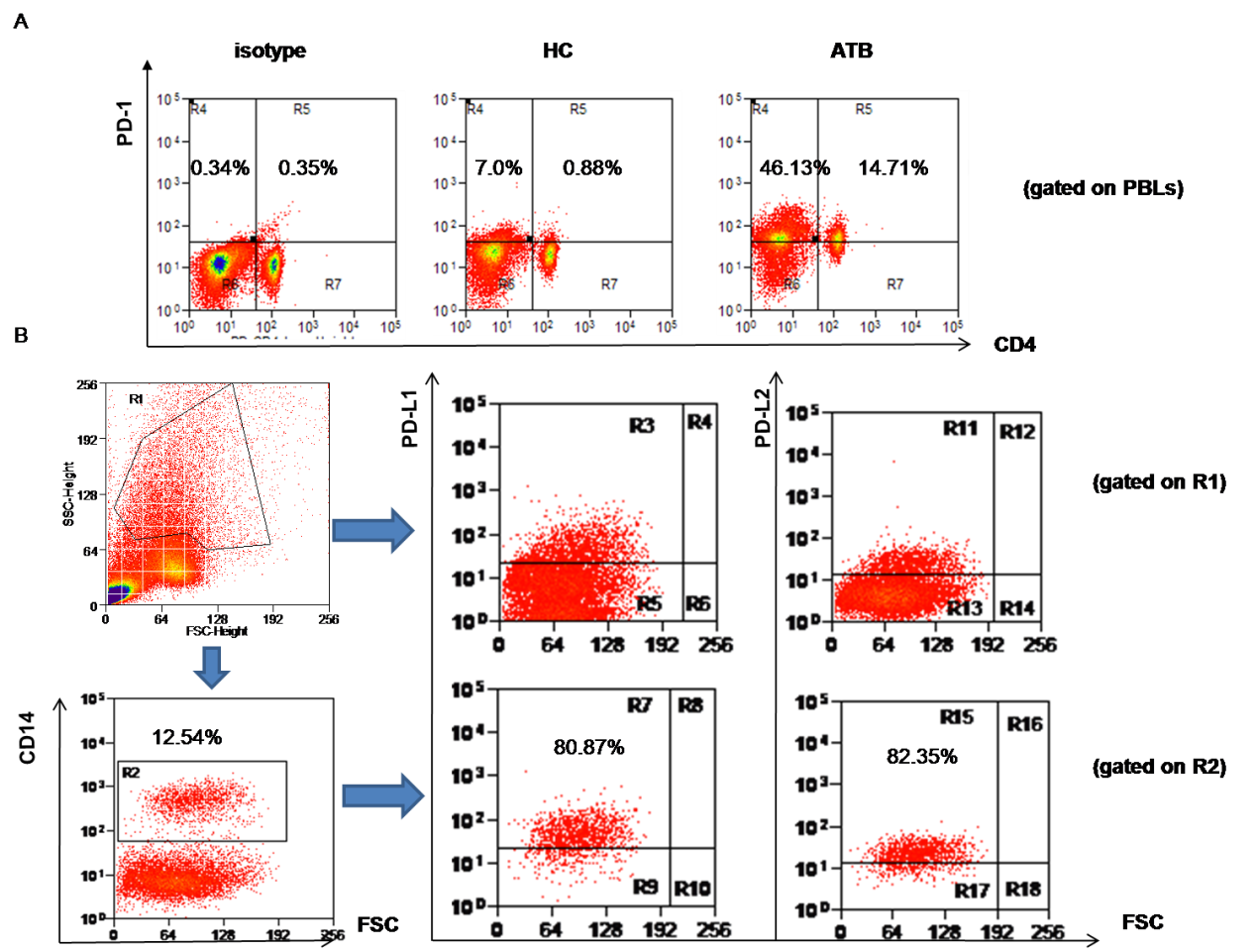
**

**Figure S1. The representative flow dot plots.** ***A*** Flow cytometry dot plots of PD-1 expression was shown gated on peripheral blood lymphocytes (PBLs). ***B*** Flow cytometry dots plots of PD-L1/L2 expression were shown gated on R1 (monocytes) and R2 (CD14^+^ monocytes).


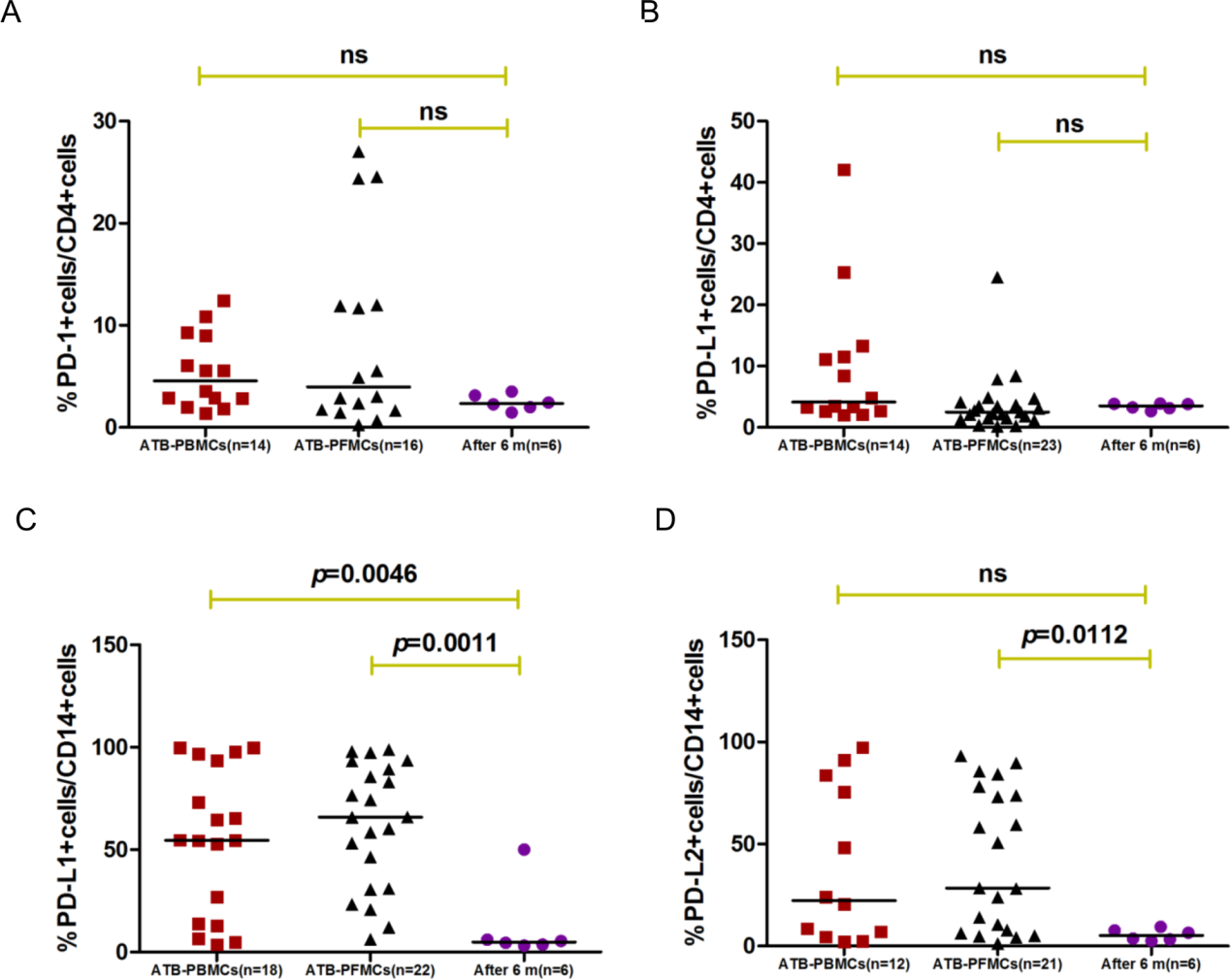


**Figure S2. The frequencies of PD-1 and its ligands on CD4^+^ T cells and CD14^+^ monocytes in ATB peripheral blood and pleural effusion, as well as in peripheral blood of patients treated for 6 months.** ***A*** Frequency of PD-1 on CD4^+^ T cells was similar before and after treatment. ***B*** Frequency of PD-L1 on CD4^+^ T cells was similar before and after treatment. ***C*** Frequency of PD-L1 on CD14^+^ monocytes in blood and pleural effusion decreased more significantly after treatment than before treatment. ***D*** Frequency of PD-L2 on CD14^+^ monocytes in pleural effusion decreased more significantly after treatment than before.


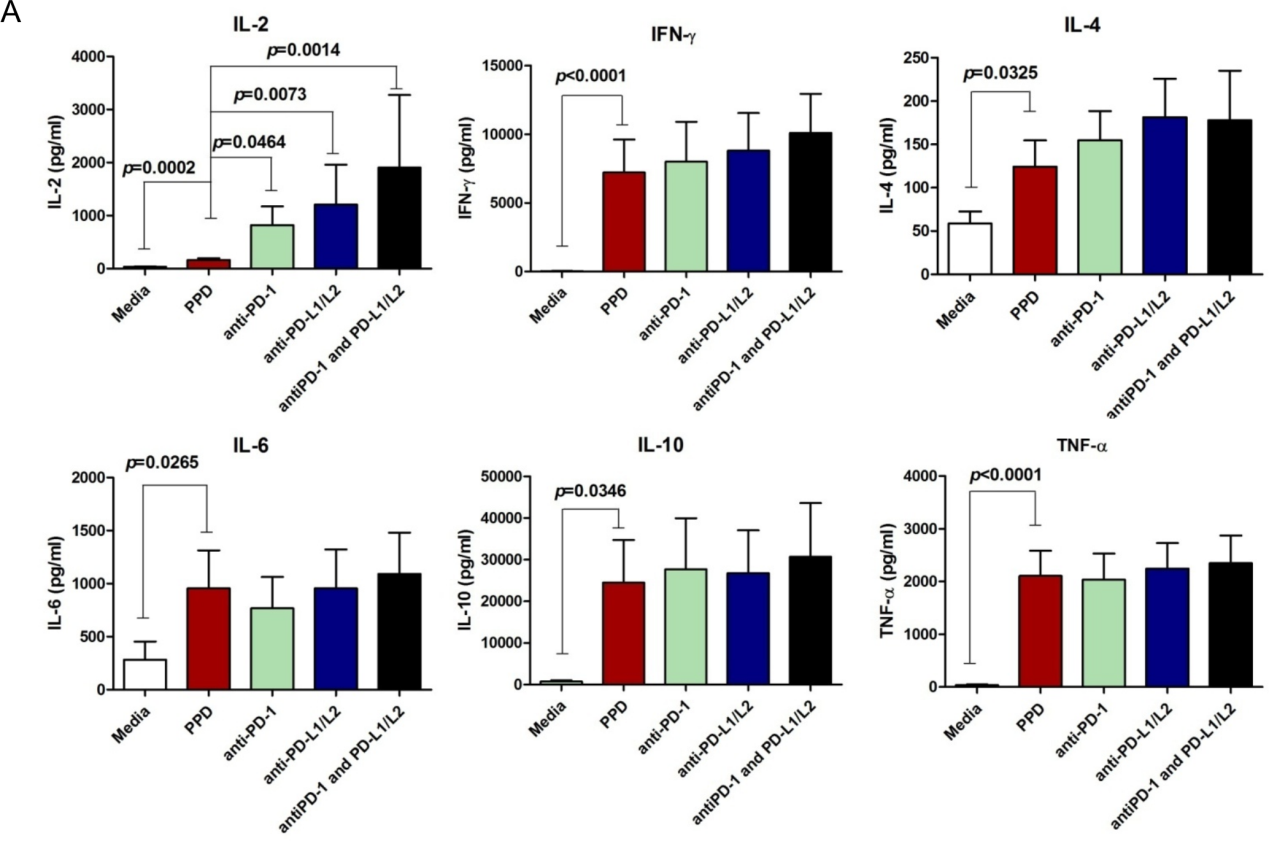


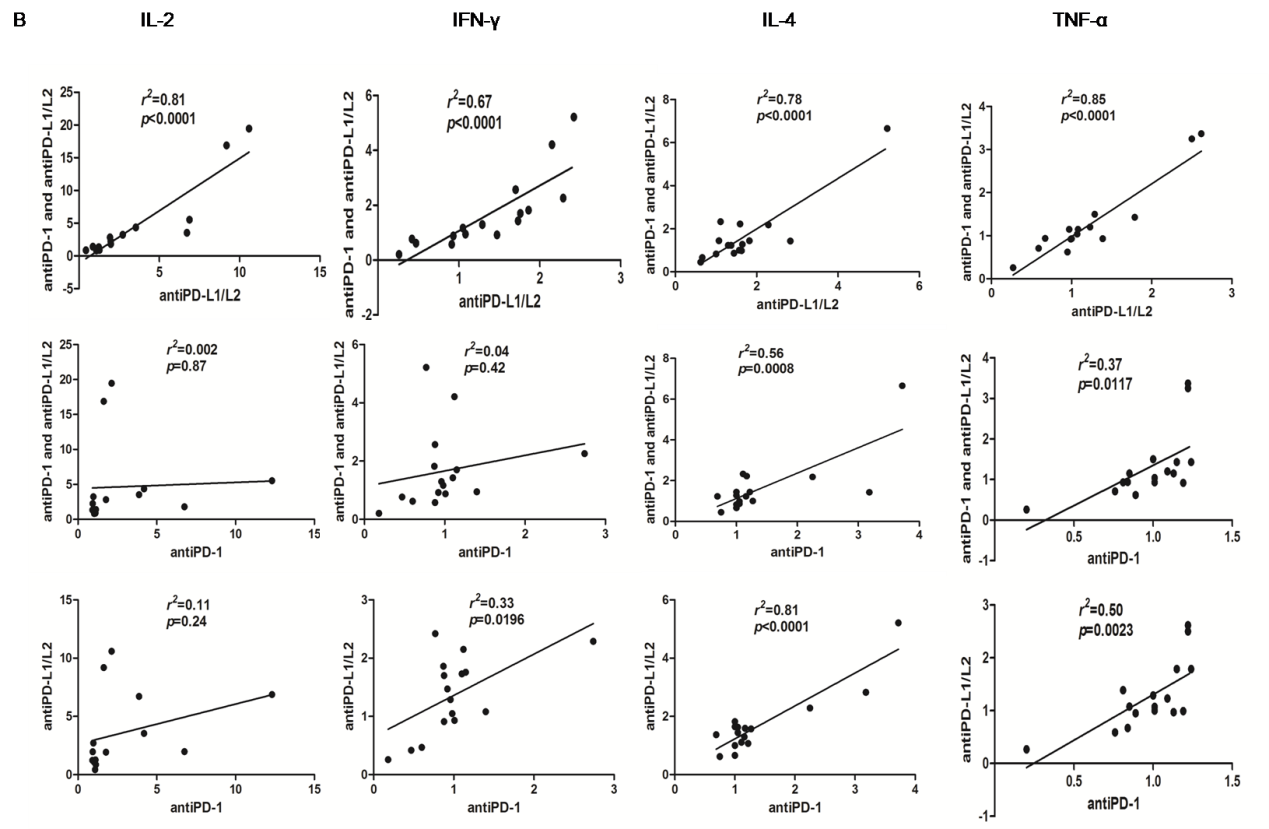


**Figure S3. Combined blockade of PD-1 and PD-L1/L2 strongly affected IL-2 levels secreted by PBMCs stimulated with PPD in active tuberculosis.** ***A*** Combined blockade of PD-1 and PD-L1/L2 resulted in a 10- to 20-fold increase of IL-2 secretion compared to PPD stimulation only. However, combined blockade of PD-1 and PD-L1/L2 did not significantly influence IFN-γ, IL-4, IL-6, IL-10 and TNF-α levels compared to PPD stimulation only. ***B*** The correlation of cytokine secretion index with PD-L1/L2 blockade. The upper row shows that PD-1 and PD-L1/L2 combined blockade is strongly correlated with PD-L1/L2 blockade including IL-2 and TNF-α. The middle row shows the correlation between PD-1 and PD-L1/L2 combined blockade and PD-1 blockade. The lower row shows the correlation between PD-L1/L2 blockade and PD-1 blockade. All the x-axis and y-axis were symbols of cytokine secretion index. The anti-PD-1 and anti-PD-L1/L2 secretion index was the ratio of cytokine secretion level when blockade with PD-1 and PD-L1/L2 compared to PPD stimulation only. The anti-PD-L1/L2 secretion index was the ratio of cytokine secretion level when blockade with PD-L1/L2 compared to PPD stimulation only. The anti-PD-1 secretion index was the ratio of cytokine secretion level when blockade with PD-1 compared to PPD stimulation only.


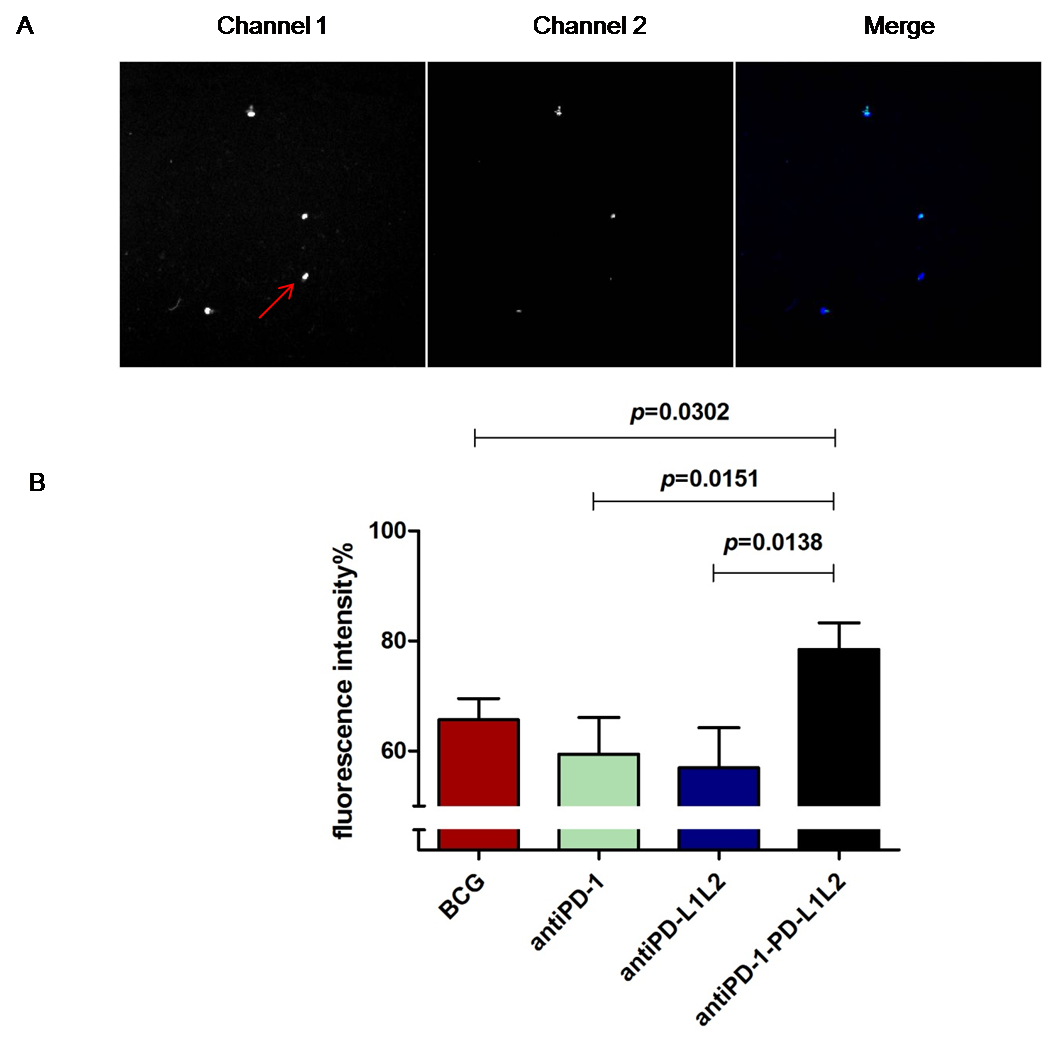


**Figure S4. Combined blockade of PD-1 and PD-L1/L2 enhanced the macrophage phagocytosis of BCG. *A*** Fluorescence microscope scan of BCG phagocytosis by macrophages. Macrophages were scanned by channel 1 displayed as a blue signal in the merge view. BCG was scanned by channel 2 displayed as a green signal in the merge view. The red arrow in channel 1 represents the macrophage which does not phagocytize the BCG. ***B*** A significant increase in the percentage of phagocytosis in the presence of both PD-1 and PD-L1/L2 blocking Abs was observed compared with that without blockade or mono-antibody blockade (*P*=0.0302, *P*=0.0151, and *P*=0.0138, respectively).
